# Supplementary material for: Are carbon emissions trading and green financial instruments synergistic? -Comprehensive quantitative research based on content analysis
Source: PLoS One. 2024 Mar 7;19(3):e0298601. doi: 10.1371/journal.pone.0298601 (PMC10919601; doi:10.1371/journal.pone.0298601)
Supplement: S1 Data — (ZIP) [file pone.0298601.s001.zip › Data/地级市Z_F工作报告.zip!/░▓╗╒/┬φ░░╔╜╩╨/└┤╫╘╣1⁄2╓┌║┼í╛┬φ┐╦╩2╛▌═°í┐_2004.docx]

# **政府工作报告（2004）**

【字体大小：[大](http://zwgk.mas.gov.cn/openness/detail/content/javascript:doZoom(18)) [中](http://zwgk.mas.gov.cn/openness/detail/content/javascript:doZoom(15)) [小](http://zwgk.mas.gov.cn/openness/detail/content/javascript:doZoom(14))】

——2004年1月5日在马鞍山市第十三届人民代表大会第三次会议上

市   长    姚玉舟

各位代表：

现在，我代表市人民政府，向大会作工作报告，请予审议，并请市政协委员和其他列席人员提出意见。

一、2003年工作回顾

刚刚过去的2003年，是很不寻常、非常难忘的一年。一年来，面对非典疫情等不利因素的严峻考验，在省委、省政府和市委的坚强领导下，市政府团结和带领全市人民，锐意进取，攻坚克难，较好地完成了市十三届人大一次会议确定的各项目标任务，实现了本届政府工作的良好开局。

（一）经济发展呈现全面提速增效的良好局面。通过狠抓优化结构和促进增长的各项工作，国内生产总值、固定资产投资、社会消费品零售总额、财政收入等主要经济指标增幅跃居全省前列。全年完成国内生产总值188.5亿元，比上年增长14%。固定资产投资实现历史性跨越，首次突破百亿元大关，完成105亿元，增长70%；马钢250万吨热轧薄板等24个重点生产型项目相继建成投产，有力拉动了经济的即期增长，积蓄了发展后劲。全市财政收入在2001年突破20亿元的基础上，去年超30亿元，比上年增长29%，总量进入全省前三位。

工业经济的速度和效益大幅度提升。去年实现全部工业总产值187亿元，增长22.2%；规模以上工业实现利润30亿元，居全省各市之首；综合效益指数175，比上年提高60个百分点；新增规模以上工业企业27户。马钢生产经营创历史最好水平，钢产量跨上600万吨台阶，销售收入突破160亿元，继续发挥经济增长的支撑作用；星马、山鹰、圣戈班、中橡等骨干企业强势增长，县区工业蓬勃兴起，促进了全市工业的加快发展。十七冶坚持不懈地推进改革、强化管理、开拓市场，开始摆脱困境，产值和效益大幅度回升。设计院、矿院、安工大的院校办产业发展迅速。商贸流通业改制重组和开放引进的活力进一步显现，实现社会消费品零售总额46.8亿元，增长13.3%，是近五年来首次出现的两位数增幅。农业生产面对自然灾害的影响，种植业产值略有下降，养殖业规模进一步扩大，产业化水平迅速提高，黄池食品和雪润2户企业销售收入突破亿元。民营经济加速崛起，呈现出发展规模不断扩大、外来投资快速增长、生产型科技型企业比重明显上升的势头，在国内生产总值中的比重达到23%，比上年提高2个百分点。金融业稳健运行，对企业改革和经济建设的支持力度加大，年末银行存贷款余额分别比年初增加46.3亿元和52.8亿元。

（二）对外开放向全方位、多层次、宽领域快速拓展。去年把招商引资作为重中之重的工作，强力推行一岗双责、强化目标管理、开展系列招商活动，取得了显著成效。全年新批外资企业27户，总投资2.3亿美元，合同利用外资8109万美元，实际利用外资8000万美元，增长90%；实际利用省外资金20亿元，增长150%，增幅均居全省前列。工业招商引资迈出新步伐，法国圣戈班集团全额收购合资企业中方股份，并增资1000万美元新建大口径管件生产线；浙江万马、南通瑞慈分别在开发区投资1.3亿元和1亿元新建两个工业园。马钢对外合作取得积极进展，全年建成和新成立合资企业5户。农业招商引资实现新突破，引进“三资”农业项目108个，利用外来资金折合人民币2亿元。商贸流通业招商引资继续扩大，上海农工商、浙江海外海等国内知名企业相继进入。公用事业招商引资取得新成绩，自来水公司与首创集团、公交公司与南京中北集团全面合作正式签约，燃气总公司与香港中华煤气合作成功，与西班牙华龙公司合作建设城市管网项目已经实施，城市建设和房地产招商引资12.4亿元。

园区建设全面快速推进。通过连续高强度的投入，开发区建设取得重大进展，进入了项目大规模入区建设的新阶段，当年新批入区项目42个，总投资折合人民币27亿元；全年实现工业总产值20亿元，增长53.8%。慈湖工业园主干道路和新材料产业园建设正式启动，10个在建项目总投资折合人民币26.6亿元。当涂工业园起步区主干路网基本建成，14个项目正在抓紧实施。各区工业、旅游园基础设施建设和招商引资工作取得新成果。6个重点乡镇工业集中区入区企业达318户。

对外经贸合作与交流进一步扩大。全年完成进出口总额8亿美元，是上年的2.3倍，取得总量全省第二、增幅全省第一的新成绩，其中出口1.1亿美元。外经工作继续拓展，境外劳务输出规模不断扩大。对外友好交流与合作稳步推进，与澳大利亚科克拉市签订了缔结友好城市协议书。

区域经济合作迈出重要步伐。全面启动与南京市的交流与合作，两市签署了经济社会发展全面合作框架协议和7个专项合作协议。以承接产业梯度转移为重点，积极融入长三角，全年引进的300万元以上130个内资项目中，江浙沪占75%。与芜湖、铜陵就联手打造马芜铜现代化城市群进行了深入接洽，并在劳动就业、人才开发一体化上取得实质性进展。

（三）以企业改革为中心的改革攻坚取得历史性突破。2003年是我市企业改革攻坚年，通过强化领导、明确责任、深入发动等一系列措施，强力推进工交、城建、粮食、商贸等系统和县区各类企业的改革攻坚。按照“三个置换、一个保障”的总要求，坚持企业改革与招商引资相结合，在化解历史包袱、置换职工身份、盘活存量资产上下功夫，使企业通过改制焕发新的生机和活力。坚持因企制宜、一企一策，加强组织协调和政策指导，采取财政支持、债务重组、土地资本运营等多种形式筹措改革成本6亿多元，在养老、失业、医疗保险等方面不折不扣地维护和兑现改制企业职工的合法权益，有力地保证了全市企业改革的顺利进行。全年共完成国有和集体中小企业改革207户，超额完成年初确定的企业改革任务。提前一年实现下岗职工由基本生活保障向失业保险的转轨并线，全市201个再就业服务中心完成历史使命后顺利关闭。通过改革，全市经济结构明显优化，非公经济比重由上年的35%提高到42.4%，经济发展活力进一步增强。企业进入资本市场融资实现新突破，星马公司在上交所挂牌上市融资2.97亿元，山鹰公司2.5亿元可转换债券成功发行，天源科技公司等上市资源培育情况良好。

其他各项改革扎实推进。行政管理体制改革继续深化，对区放权工作逐步完善，再次取消行政审批项目218项。农村税费改革不断深入，农业特产税改征农业税工作扎实开展，粮食补贴方式改革试点工作全面推行，粮补资金足额兑现到户。乡镇区划调整工作稳步推进，村级债务化解工作取得积极成果。户籍管理制度改革深入实施，全年新增进城落户1.1万人。住房制度改革向深层次推进，土地资本运营力度明显加大，实现土地收益大幅度增长，房地产市场和矿业权市场通过治理整顿逐步规范。与国家开发银行达成80亿元信用合作额度，有效保障了重点企业发展和城市建设的大量资金需求。

（四）城乡建设投入之多、成效之大创历ascii-font-family: ’Times New Roman’">着眼于建设“双百”现代化大城市，完成了新一轮城市总体规划修编，已获省政府批准；对城市中心区、开发区南区、采石风景区、大学城区和采石河、慈湖河等区域性控制性规划以及部分专业规划进行了编制和评审。全年完成城市建设投资31亿元，增长59%。道路建设提前完成年度目标，城市南部的湖东南路、中心大道、九华路、印山路等13条骨干道路以及相配套的6座桥梁全部竣工，东部的湖南东路、湖北路东段、林里路一期等道路工程顺利建成，全年新增主干道路22公里，城市“东扩南进”构架初步形成。公用设施建设进一步加快，天然气利用工程基本竣工，第四水厂和第二污水处理厂加紧建设。供电、邮政等公用基础设施进一步完善，新建的慈湖市话分局正式投入使用。房地产业持续发展，全年完成开发总投资15亿元，施工面积180万平方米，竣工82万平方米。围绕建设“生态马鞍山”，积极实施退耕还林、城乡绿化美化工程，建成朱然文化公园等园林景观，顺利通过国家园林城市复查。采石风景区被评为国家4A级风景旅游区。小城镇建设取得新进展，当涂县城面貌发生明显变化，博望等重点镇的配套服务功能得到提高。马芜高速公路建设按计划推进。当高大桥主体工程基本完工，江心乡柏油路顺利建成，围乌路路基工程按计划实施，编制了大公圩道路规划，全年改扩建农村道路154公里，农村交通条件进一步改善。江堤加固、姑溪河清淤等水利国债工程全面竣工，共加固堤防54公里，疏浚河道18公里，建成3座沿江排涝站；大公圩、团结圩等万亩以上圩口堤防除险加固和沿江山丘区抗旱工程全部竣工，城乡防洪保安、抗旱减灾能力显著增强。环境卫生综合整治取得新成效，全年共拆除违法建筑15万平方米，对团结广场等城市主要路口交通拥堵状况进行了治理；创建国家环保模范城各项任务得到落实，市容市貌有了新的改观。

（五）人民生活水平继续得到较大幅度提高。坚持把促进就业再就业摆在突出位置来抓，认真贯彻落实社保补贴、岗位补贴、免费培训、岗位援助、税费减免、小额贷款等促进再就业的扶持政策，引导和鼓励各类用工单位吸纳就业，帮助下岗失业人员自谋职业、自主创业。支持总工会、共青团、妇联开展结对帮扶、青年创业、巾帼创业等就业援助活动。着力加强就业再就业市场服务体系建设，完成城乡就业再就业和养老保险信息联网工程，在全省率先建成街道和社区劳动保障工作平台。全年新增就业岗位2.2万个，实现就业再就业1.4万人，城镇登记失业率为4.2%。把扩大农村劳务输出作为农民增收的重要举措，强化组织和服务，农村劳务输出规模达到13.2万人。社会保障体系进一步完善，城镇养老、失业、医疗保险健康运行，其中医疗保险参保人数扩大到26.2万人，覆盖率达95%，居全省首位。企业退休人员社会化管理服务工作开始实施。城市低保工作成果进一步巩固，并实现动态管理。社会捐助和扶贫济困活动深入开展。实施了廉租房制度，按全省最高标准对低保住房特困户实行住房补贴。完成背街小巷和老旧小区综合整治二期工程，启动首批成片危旧房改造项目，群众生活环境继续改善。“星光计划”实施取得突破性进展，完成了74个社区的办公及老年人活动设施建设，街道社区工作条件和工作水平明显提高。年初安排的8件为民办实事项目已完成6件，2件按计划推进。城乡居民收入大幅度增长，城镇居民人均可支配收入8750元，增长13.3%；农民人均纯收入3090元，增长6%。城乡居民人均储蓄接近1万元，净增2160元。

（六）各项社会事业和精神文明、民主法制建设全面加强。科技工作取得新成果。国家“863”计划新材料成果转化及产业化基地获准在我市建设，规划编制工作已经展开，有10个项目进入国家级科技计划。通过举办产学研合作洽谈会等多种形式，促成企业与科研院所达成合作协议70余项。“数字马鞍山”建设迈出重要步伐，城市“一卡通”系统在全国中等城市中率先建成投入使用，政府网、校园网、企业网等网络资源得到整合，电子政务、网上招商、农村信息网显示出良好的应用前景。教育事业发展加快。多元化筹资力度加大，全年完成教育投资近5亿元，创历史最好水平。地方高等教育发展取得新突破，安徽冶金科技职业学院成功组建，师范学校新校区一期工程建成使用，网络大学园一期工程正在加紧建设，安徽工贸技师学院开工建设。基础教育办学水平进一步提高，当涂一中进入省示范高中行列，市二中迁址新建工程加紧推进，十七冶自办中小学分离工作顺利完成，当涂县中小学2万平方米D类危房改造任务已经完成。由上海建平教育集团等单位投资管理的中加双语学校实现当年建设、当年招生。引进人才和国外智力取得积极成果，首次面向全国招收硕士研究生9名，组织实施引智项目10个。城乡医疗卫生工作迈出新的步伐，医疗机构体制改革正式启动，疾病预防控制中心开工建设，传染病医院和紧急救援中心加紧筹建。文化、体育、广播电视事业不断发展，丰富了广大人民群众的精神生活。成功举办了“江南之花”企业文化艺术节和第二届社区运动会，组团参加第五届全国城市运动会和第八届“国际奥委会主席杯”全国自行车赛总决赛取得较好成绩，中国国际吟诗节在创新形式和扩大群众参与面上进行了积极探索。人口与计划生育工作得到加强，人口出生率控制在省下达的指标内。《马鞍山市志》续修工作开始启动。文明创建工??验收，并荣获“全省创建文明县城工作先进县”称号。以打造“信用马鞍山”为主题的公民道德建设不断深入，市民素质进一步提高。法制宣传教育工作稳步推进，社会治安综合治理的各项措施得到有效落实。信访工作为化解社会矛盾发挥了重要作用。仲裁工作在解决经济纠纷方面作出了积极贡献。安全生产形势保持稳定。人民武装、国防动员协调发展，“双拥”和人防建设继续加强，当涂县连续三年荣获“全国双拥模范县”称号。民族宗教工作稳步发展，其他各项事业都有新的进步。

一年来，市政府自觉接受市人大和市政协的监督，政府系统承办的8件人大代表议案、123件建议批评意见和341件政协提案如期办复。完善了政府议事规则和决策机制，实行重大事项公示制度，推进了决策科学化、民主化进程。依法治市、依法行政工作不断加强。以完善目标管理为重点的工作责任体系进一步健全，行政效率继续提高。通过开展机关作风建设，政府系统的工作作风有了较大改进。行政监察工作深入开展，廉政建设责任制得到落实。

在全面总结2003年工作的时候，特别令人难忘的是，在人民群众身体健康和生命安全遭受非典威胁的严峻形势下，我们按照“防范得严、发现得早、控制得住、处理得好”的要求，迅速建立起高效运转的组织指挥体系和坚强牢固的预防控制网络，依法实施科学的防治措施，夺取了抗击非典整体战的阶段性重大胜利。在这场没有硝烟的特殊战争中，全市人民万众一心，众志成城，不怕困难，顽强拼搏，显现出空前的凝聚力和战斗力，为我们战胜前进道路上的各种困难积累了宝贵的精神财富。

回顾过去一年的政府工作，我们始终坚持以邓小平理论、“三个代表”重要思想和党的十六大精神为指导，解放思想、实事求是、与时俱进，以求新思进的积极姿态，谋大事、抓实事，开拓进取、奋勇争先；始终坚持把发展作为第一要务，紧紧围绕经济建设这个中心，全力组织招商引资、企业改革、重点建设、农村发展等重点工作的新突破，凝心聚力求发展，千方百计增后劲；始终坚持统筹兼顾、可持续发展，在大力推进物质文明建设的同时，高度重视政治文明、精神文明建设，在城市规划的修编与执行、环境的建设与保护、计划生育与提高人口素质等方面，充分体现和落实城市长远发展的要求，促进人与自然的协调发展和社会的全面进步；始终坚持以人为本、执政为民，把不断改善人民生活作为政府工作的根本出发点和归宿，在推进改革和建设的过程中，充分考虑绝大多数人的利益和意愿，实施最优惠的医保、住房补贴、城市低保、再就业扶持等政策，着力解决特困群体的生产生活问题，努力实践人民政府的根本宗旨。

各位代表，2003年的成绩来之不易、令人鼓舞，这是市委正确领导、市人大市政协监督支持、全市人民团结奋斗的结果。在此，我代表市人民政府，向全市广大工人、农民、知识分子、干部，向市人大代表、政协委员，向各民主党派、工商联、无党派人士和群众团体，向中央和省驻马单位、来马投资企业，向驻马部队官兵和政法干警，向所有关心支持我市建设和发展的同志们、朋友们，致以崇高的敬意和衷心的感谢！

我们也清醒地看到，我市经济和社会发展还面临不少困难，工作中还存在一些不足。一是经济结构性矛盾仍然比较突出，传统产业竞争力不强，民营经济和开放型经济实力较弱，把城市做大做强做优的任务依然繁重。二是促进农民收入持续增长的难度较大，城乡就业再就业矛盾较为突出，部分群众的生活还比较困难。三是经济发展环境有待进一步优化，城市长效管理机制尚需完善，政府职能转变和机关作风建设与形势发展要求还有一定差距。我们将高度重视这些问题，下大力气逐步加以解决。

二、今后一个时期的发展目标与2004年主要任务

在党的十六大和十六届三中全会精神指引下，全国各地发展热潮不断升温，东部沿海地区尤其是长三角城市进入全面提速、跨越奋进的新时期，省内城市加快发展、奋力赶超的举措纷纷出台。面对千帆竞发的新形势，市委六届五次全体会议及时作出了在全省率先全面建成小康社会、率先基本实现现代化的重大决策。这不仅是省委、省政府对马鞍山的要求和期望，也是全市人民的根本利益所在。政府工作要通过“三步走、翻三番”，确保“两个率先”目标的圆满实现。第一步到2006年，全市GDP比2000年翻一番，人均GDP超2500美元，财政收入与GDP保持同步增长，各项社会事业协调发展，为全面建成小康社会打下坚实的基础。第二步到2012年，实现GDP比2000年翻两番，人均GDP达5000美元，城镇居民人均可支配收入达18000元，农民人均纯收入达7600元，政治文明、精神文明协调发展，提前八年在全省率先全面建成小康社会。第三步到2018年，实现GDP比2000年翻三番，把马鞍山建设成为经济繁荣、环境优美、生活富裕、社会文明的城市，在全省率先基本实现现代化。

今年是实施“两个率先”战略的重要一年。当前，世界经济逐步回升和结构调整步伐加快，国际资本将继续大举进入我国，为我们进一步扩大对外开放提供了新的契机；我国经济发展正处于周期性的上升阶段，新一轮消费结构升级的趋势明显，将有力推动我市产业结构的优化调整；多年积累的改革开放成果以及一批重大项目的建成发挥效益，为我们加速发展奠定了坚实的物质技术基础。同时我们也看到，由于国际环境不确定因素较多，加上国家对土地、金融、税收等重要政策进行一系列调整，由此带来影响的范围和程度还很难预料，今年的发展将面临新的挑战。我们必须兴利除弊，超前谋划，以更大的力度、更有效的举措，奋力开创经济社会发展新局面。今年政府工作的指导思想是：以邓小平理论和“三个代表”重要思想为指导，全面贯彻党的十六大和十六届三中全会精神，坚持以人为本，树立全面、协调、可持续的发展观，以“两个率先”统揽工作全局，进一步加快改革开放步伐，加大结构调整力度，协调推进“三个文明”建设，不断提高人民生活水平，努力实现经济持续快速协调健康发展和社会全面进步。全市经济和社会发展的主要预期目标为：国内生产总值增长12%，财政收入基本同步增长，固定资产投资增长20%以上，社会消费品零售总额增长8%，城镇居民人均可支配收入和农民人均纯收入分别增长9%和5%，新增就业岗位2万个，城镇登记失业率控制在4.5%以内，人口出生率控制在省下达的指标内。为此，必须努力做好以下九个方面的工作。

（一）深入推进各项改革，有效激发经济社会发展的活力。把今年作为深化改革年，认真贯彻《中共中央关于完善社会主义市场经济体制若干问题的决定》，加快推进改革攻坚，进一步解放和发展生产力，为全市经济社会协调发展增添动力。

基本完成企业改革攻坚任务。在巩固企业改革成果的基础上，进一步强化责任，全面完成国有、集体中小企业改革任务，推动大企业改革取得新突破。重点抓好商贸流通企业改革，推动企业实现开放式重组。加快盘活破产关停企业的沉淀资产，培育新的经济增长点。通过规范上市、相互持股、民营参与等多种形式，发展国有资本、集体资本和民间资本参股的混合所有制经济，实现股权多元化。支持马钢集团公司深化产权制度改革，逐步建立国有控股或相对控股的多元股权结构。支持十七冶、设计??动，不断培育和壮大优势产业。全面完善乡镇企业改革。继续推进国有资产管理体制改革，积极探索行政事业单位国有资产管理的有效途径，建立国有资产经营责任制度，实现国有资产保值增值。

大胆探索投融资体制改革。坚持“谁投资、谁决策、谁收益、谁承担风险”的原则，放宽投资领域，强化企业投资主体地位。积极拓展企业融资新渠道，鼓励上市公司通过资本市场再融资，支持天源等优势骨干企业如期上市。进一步完善政府投资体制，将经营性和准经营性项目逐步推向市场，鼓励和引导社会资本以多种方式参与建设。规范政府投资项目决策程序，落实责任主体，强化投资管理，进一步提高政府投资效益。

积极推进事业单位改革。按照“脱钩、放权、分类、搞活”的总体要求和“先易后难、循序渐进、逐步推开、稳步实施”的原则，根据事业性质和单位情况，研究政策，积极尝试，力争在经营性事业单位的企业化改制上取得新的突破。实施事业单位人事制度改革试点，在教育等事业单位实行全员聘用制，推行竞争上岗，完善专业技术职务聘任制度。探索事业单位分配制度改革，逐步形成重实绩、重贡献、向优秀人才和关键岗位倾斜的多元化分配新机制。

继续深化行政管理体制改革。以优化发展环境、提高行政效率、建设服务型政府为目标，加快政府职能从管制型向服务型转变。全面完成撤乡并村工作，稳妥安置分流人员。进一步完善对区放权，理顺行政管理体制，促进区街经济更快发展。深化和完善户籍管理制度改革。深入推进行政审批制度改革，继续清理和削减审批事项，简化审批程序。加快推进电子政务建设，不断提高政府工作效率。大力推进城市社区建设，创新管理体制，拓展服务范围，提升社区自我管理、自我服务功能。

（二）全面扩大对外对内开放，推动开放型经济和民营经济加速发展。继续强力实施大开放主战略，积极开展“扩大开放年”活动，推动招商引资在更大范围、更广领域、更高层次上的突破，形成全方位开放的新局面。

全力以赴推动招商引资再上新台阶。坚持把招商引资作为经济工作的重中之重，不断强化以目标分解、督查考核、兑现奖惩为主要内容的目标责任制，进一步调动方方面面招商引资的积极性，确保全年实际利用外资8000万美元、省外资金27亿元以上。国内以长三角、珠三角和港台为重点，国外突出日韩，兼顾欧美等地，大力推进以外引外、产业链招商、代理招商、网上招商、小分队驻点招商和利用友城招商，充分发挥园区和驻外机构的作用，加快大企业、大项目利用外资步伐，鼓励引导民营企业对外招商引资。在招商内容上，注重引进一批有利于推进产业结构升级的加工制造业项目，扩大现代农业、服务业、基础设施等领域的对外开放。全面加大项目落实力度，对在建的一批外来投资项目，实行全程跟踪服务，促其尽快建成投产；对一批有意向、条件较好的合资合作大项目，盯紧不放，争取尽快签约实施。加强招商引资项目库建设，健全各项工作制度，夯实招商引资工作基础。下更大的气力改善投资软环境，努力营造亲商、安商、富商的浓厚氛围，争创招商引资新优势。

毫不动摇地推进园区建设。根据国家政策调整导向，完善各类园区发展规划，加快推进基础设施建设。开发区要在抓紧西区项目建设、展开东区项目布点的同时，大力推进采石河以南地区“二纵五横”路网建设，加速向南发展。全面展开慈湖工业园主干路网建设，尽快形成具备承载新的大型项目的基础条件。进一步加快县区工业、旅游园建设，确保符合项目布点条件的区域达到10平方公里以上。以产业分工带动项目引进，努力促进各具特色的优势产业和强势企业向园区集聚。

积极参与国际竞争与合作。深入研究落实国家出口退税新政策，充分利用中央和省里的外贸促进政策，大力开拓国际市场，培育和发展新的出口增长点，确保全年外贸出口1.2亿美元以上。进一步完善口岸体系，改善外贸通关条件。积极实施“走出去”战略，发挥马钢、十七冶等大企业外经工作优势，发展境外技术合作和工程承包，鼓励和支持各类企业开展境外加工业务和劳务输出，力争外经营业额大幅度增长。

进一步加快区域合作步伐。加紧开展市区至禄口机场高速公路、长江大桥和宁马轻轨等重大交通项目前期工作。实施宁马两市全面合作框架协议和科技、规划、农产品流通、旅游、交通基础设施建设、人事人才、劳动和社会保障等专项合作协议，全方位参与南京都市圈建设。主动承接长三角尤其是上海、苏南地区的经济辐射，实现产业发展的良性互动。继续加强与芜湖、铜陵两市的深度合作，积极参与打造马芜铜现代化城市群。

大力支持和放手发展民营经济。全面落实中央和省、市关于鼓励扶持民营经济发展的各项政策，努力消除体制性障碍。放宽市场准入领域，凡国家法律法规没有明令禁止的领域和项目，只要符合我市经济发展要求，均支持民营经济投入与经营。继续深化并联审批制度改革，全面推行告知承诺制，减少审批环节，为民营经济发展提供高效服务。大力拓宽民营经济融资渠道，支持建立民间信用担保公司和风险投资机构，构建投资主体多元化、形式多样化的民营企业信用担保体系和风险投资体系。培育一批民营企业大户，推动民营经济结构优化升级。鼓励机关和企事业单位人员离岗创业，鼓励大中专毕业生和下岗失业人员自主创业。把发展民营经济纳入政府工作目标考核体系，确保全年新发展民营企业1000户以上，民营经济增加值占国内生产总值比重达到25%，纳税额超2亿元。

（三）进一步强化工业主体地位，着力提升工业经济规模和实力。坚持走新型工业化道路，按照建设现代加工制造业基地的要求，加大技术改造和开放引进力度，全面提升制造业水平。重视研究和协调解决企业生产中的新情况新问题，促进工业经济的健康运行。

全力支持马钢和一批骨干企业的建设发展。加快马钢“十五”重大技改项目建设，确保冷轧薄板、彩涂板生产线等重点工程按期建成投产，抓好热轧薄板等项目的达产见效，力争全年产钢800万吨；扎实做好第二条H型钢生产线等重点工程的前期准备，争取尽快实施；积极拓展壮大非钢产业，加速推进主辅分离。支持马钢提前启动“十一五”500万吨钢新项目规划，完成土地征迁和场地“三通一平”，力争年底开工建设，为早日建成具有国际竞争力的现代化大型企业集团奠定基础。积极支持星马、山鹰、金星、圣戈班、中橡等骨干企业的技术改造，完成和开工建设一批新项目，大力开发新品种，不断提高市场占有率。扎实推进二电厂扩建工程建设，认真做好联合发电厂前期准备工作，加快发展电力能源产业。

推动中小企业上规模、上水平。选择一批成长性较好、管理水平较高、制度较完善的中小企业，予以重点扶持，促使其加快发展，力争全年新增规模以上工业企业30户，新增销售收入超亿元企业5户以上。组建多层次、多种所有制的融资担保体系，拓展中小企业融资渠道。通过商业银行开展的联合授信，重点加大对30—50户专精特新企业的信贷支持力度。整合和提高市级各类产业引导资金的使用效率，加强对中小企业跟踪服务，促进中小企业快速成长。

（四）以增加农民收入为核心，加快农业和农村经济发展步伐。大力发展现代农业。围绕建设有区域特色的绿色食品基地，千方百计引进外来资本、工商资本和民间资本深度开发农业资源，促进精品农业、特色农业和生态农业的加速兴起。加快农业产业化进程，重点引进和培育一批主营产品优势明显、成长潜力较大、带动农民增收能力较强的龙头企业，发展壮大产业化龙头企业群体。大力开拓水产、畜禽、粮油、蔬菜、花卉苗木等农产品的生产经营，推动优势名牌产品向优势产区、龙头企业集中；发挥能人作用，培育种养大户，扩大规模经营和品牌效益。加强农产品流通体系建设，实施优质农产品进超市工程，进一步畅通农产品营销渠道。充分发挥农产品质量安全监督检测中心作用，严格无公害农产品市场准入，促进农业标准化生产。

加快农村城镇化进程。加紧编制以中心镇为重点的新一轮乡镇发展总体规划。继续采取综合考评、以奖代补政策，推动小城镇基础设施建设实现新突破。全年完成小城镇建设投资5亿元，城镇化率提高2.4个百分点，达到56%。以产业集聚推动小城镇经济加快发展，支持重点乡镇工业集中区立足现有基础，发展刃模具、机床、金属冶炼、纺织服装、农副产品加工等产业，着力提升小城镇经济总量和吸纳农村劳动力的能力。

深化农村各项改革。继续推进农村税费、粮食补贴等改革，进一步规范农村收费行为，完善村务公开，维护农民利益。稳定和完善土地家庭承包经营责任制，支持农民按照依法、有偿、自愿的原则，采用租赁、转包、互换、入股等多种形式，实现集体土地经营权有序流转，发展适度规模经营。认真落实耕地保护制度，加强土地复垦工作。实施国有林场、乡村集体林场改制，推动小型水利工程产权制度和管理体制改革。深入贯彻党在农村的各项政策，不断加大对农业的支持和保护力度。

（五）大力发展第三产业，进一步提高现代服务业水平。下功夫培育旅游业。以打造休闲旅游基地为方向，积极实施全市旅游总体规划，全面启动采石风景区核心景区的综合开发；对城市中心景区的景观进行整合和优化，打通雨山湖环湖游道。加强旅游服务设施建设，改造新建3—5家三星级以上宾馆。高起点策划大型旅游项目和旅游活动，开发一批标志鲜明、各具特色的旅游产品。加快推进旅游业管理体制和经营机制创新，采取特许经营、出让经营权等多种形式，把优质旅游资源推向市场，增强旅游业发展的活力和动力。积极开辟宣传促销渠道，努力扩大旅游消费市场。全年实现旅游业总收入10亿元以上。

促进房地产业持续健康发展。全年房地产开发投资力争完成20亿元，比上年增长30%以上，施工面积180万平方米，其中新开工100万平方米，竣工90万平方米，建成一批不同档次的住宅小区，完善住房供应体系，满足居民多层次的消费需求，使城市人均住房建筑面积达到22平方米。加大政策、资金扶持力度，继续实施市区成片危旧住房改造工程，力争完成17万平方米危旧住房改造任务。进一步规范和加强房地产市场管理。全面贯彻《物业管理条例》，不断提高物业管理水平。

加快发展商贸流通业和金融业。积极引进外资外企重组我市商贸流通企业，鼓励和支持民间资本参与商贸流通领域的改革和发展，加快发展购物中心、现代连锁等新型业态，努力提升商业的规模和档次。加快建设商业步行街，完善解放路商业街功能，全面启动新源购物中心建设，超前规划建设集贸市场和小商品零售市场，支持发展社区商业网点。加大金融安全区创建力度，推进城市商业银行和农村信用联社深化改革，支持金融机构开展业务创新，探索发展多种所有制形式的金融企业，促进金融业更快发展。继续发展会计、律师、咨询等中介服务组织。

（六）以更大力度抓好城乡建设，促进“双百”城市构架的加快形成。加大基础设施建设力度。继续推进城市主干道路建设，新建慈湖河路、霍里山大道等6条道路，延伸湖南东路、印山路、花园路、林里路等9条道路，新增道路总长30公里。加快湖西路与314省道连接，打通与当涂县城的快速通道，为全面实施城市“东扩南进”战略创造条件。加强农村交通设施建设，建成当高大桥，完善以大公圩为核心的县乡道路规划，做好建设黄池大桥的前期工作，新建农村道路100公里，带动农村经济加速发展。进一步完善城市公用设施，天然气利用一期工程发挥使用效益，建成第二污水处理厂，加快第四水厂、第一污水处理厂、向山垃圾场垃圾无害化处理工程建设进度，不断提高城市配套服务功能。实施背街小巷和老旧小区综合整治三期工程，改造33条背街小巷道路和8个老旧小区，促进市容市貌的进一步改观。组织实施石臼湖堤防应急加固、小水库除险加固、城市防洪圈堤加固等重点工程，坚持开展水利兴修，不断增强城乡防洪保安和抗旱减灾能力。

加强“生态马鞍山”建设。抓紧制定生态园林城市建设规划，启动采石河综合整治工程，建设佳山、雨山公园，彻底整治雨山湖，开工建设市中心广场，打造城市建设精品，提升城市品位。开展慈湖河综合整治的规划设计工作，为早日实施创造条件。以工业污染防治为重点，全面完成创建国家环保模范城各项基础性工作，迎接国家验收。加强农村环境综合治理，突出抓好农村水资源保护和饮用水管理，积极支持当涂县做好大公圩水环境综合整治工作，促进农业生产条件和农民生活质量的进一步改善。

强化城市规划、管理和经营。认真执行新一轮城市总体规划，加快推进分区规划和专业性规划编制。加强城市东部地区的规划管理。完善落实长效管理的各项措施，重点加强对违法建筑、乱倒垃圾、噪声废气污染等影响市容和居民正常生活的重难点问题的整治，不断提高城市管理水平。进一步规范和完善国土资本运营工作，最大限度地发挥土地资源的效能。加大经营城市力度，积极探索以市场化方式运作基础设施的建设和管理。

（七）统筹做好城乡就业再就业和社会保障工作，不断改善人民生font-family: ’Times New Roman’">千方百计促进就业再就业。把扩大就业和再就业作为政府的一项重要工作，继续认真贯彻就业优先方针，积极推进就业体制改革，健全就业服务体系，加强职业技能培训和再就业培训，不断提高劳动者的择业竞争能力。认真落实再就业扶持政策，继续做好《再就业优惠证》发放工作，加大对下岗失业人员自谋职业、自主创业和组织起来就业的扶持力度，大力开发社区就业岗位和公益性岗位，支持发展就业容量大的劳动密集型企业，鼓励和支持多种形式的灵活就业。采取更加有力的措施，帮助大龄就业困难对象实现再就业。支持总工会、共青团、妇联等群团组织开展扶持创岗创业和再就业援助活动。高度重视并切实做好大中专毕业生的就业服务。加强劳动合同管理，维护用工双方的合法权益。严格执行国家支持和保护农民进城务工的政策规定，建立健全农村劳务输出管理机构，强化就业指导和跟踪服务，提高劳务输出组织化程度，力争全年新增农村劳务输出2万人。

进一步健全社会保障体系。以吸纳私营企业、自谋职业者、新办企业职工参保为重点，努力扩大城镇养老、失业、医疗保险覆盖面，各项社会保险基金征缴率保持在92%以上。实施企业职工工伤保险制度改革。继续推行企业退休人员社会化管理服务工作，确保社会化管理服务率达90%以上。稳步推进农村养老保险制度改革，提高农村社会保障水平。全面落实最低生活保障制度，解决城市特困群体的生活困难。积极发展慈善事业，完善社会救助机制，整合社会救助资源，倡导志愿者活动，引导社会力量支持救助事业。健全街道、社区就业和社会保障工作机制，提高街道社区工作水平。

着力解决事关群众切身利益的重难点问题。积极开展土地征用制度改革，加强和完善城市房屋拆迁管理，妥善解决征迁安置和费用补偿问题，切实保障征地农民和城市拆迁户的合法权益。继续实施廉租房制度，完善住房公积金制度，兑现低保特困户的住房补贴政策，解决城镇低收入群众的住房困难。新建改造一批老年公寓和敬老院，改善老年人的生活条件。

（八）强力实施科教兴市战略，努力构建综合竞争新优势。积极推进科技进步与技术创新。加快国家“863”计划新材料成果转化及产业化基地和科创中心后续工程建设，开工建设新材料产业园核心区、新材料孵化园等重点工程，促进高新技术产业化。继续实施“数字马鞍山”工程，加大信息基础设施建设力度，下功夫发展信息产业，大力推广信息化应用。支持支柱产业、重点行业和大中型企业建立技术中心，集中力量在关键领域抓好科技成果的中试和二次开发，不断提高企业核心竞争力和成果转化能力。深入推进产学研合作，鼓励企业加强与知名高等院校和科研院所建立紧密型合作关系，实现在合作方式、内容上的创新。进一步深化科技体制改革，制定实施《马鞍山市科技奖励办法》，鼓励和支持科技人员、社会力量领办和创办科技型企业。依法开展科普工作，大力普及科学文化知识。

努力提高教育质量和办学水平。全面推进素质教育，着重培养学生的社会实践能力和创新精神，增强教育服务经济建设、促进社会发展的功能。巩固提高九年义务教育和高中阶段教育成果，扩大优质教育资源总量，完成市二中迁址新建工程，加快建设师苑小学，力争安工大附中、市二十二中进入省示范高中行列，稳步实施马钢自办中小学分离工作。大力发展职业教育，基本建成安徽工贸技师学院。加快高等教育发展步伐，全面展开大学城建设，实施安工大东校区二期工程，完善网络大学园功能，争取申办师范专科学校通过教育部评审。进一步完善多渠道筹措经费的教育投入体制，形成公办学校和民办学校共同加快发展的新格局。认真落实市委关于建设学习型城市的决定，积极创建各类学习型组织，形成人人终身学习的浓厚氛围。

加强人才资源开发和利用。适应经济和社会发展的需要，创新人才引进政策和制度，全年力争引进400名中级职称以上的急需人才；加强对电子政务、网络管理和经营管理等各类人才的教育和培养；不断完善用人机制，充分发挥各类人才的特长和优势。加快人才市场信息化进程，全面提升人才市场服务功能和人才配置功能。以产学研、国企改革等重点项目为依托，继续加强国外智力引进工作。

（九）协调发展各项社会事业，推动精神文明建设再上新台阶。加快各项社会事业的改革和发展。深化医疗卫生体制改革，建成市疾病预防控制中心和紧急救援中心，开工建设市传染病医院，不断提高公共卫生突发事件应急处理能力。全面推进农村卫生事业发展，加强农村公共卫生设施和医疗队伍建设，积极推行以大病统筹为重点的新型农村合作医疗制度改革试点工作。进一步深化文化体制改革，大力发展文学艺术、社会科学、新闻出版等事业，加大文物保护力度，促进文化事业和文化产业协调发展。加强语言文字工作，不断提高用语用字规范化水平。加快发展广播电视事业，推进数字电视工程。积极申办首届中国国际诗歌节，丰富国际吟诗节的内涵。以市场化投入为主体，开工建设大剧院、博物馆和新图书馆。广泛开展全民健身运动，推动体育进社区、进企业。继续加强人口与计划生育工作，稳定低生育水平，促使出生人口性别比的平衡。认真开展第一次经济普查工作。扎实做好民族、宗教、对台、侨务等各项工作，切实维护妇女、儿童、老年人的合法权益，促进残疾人事业的新发展。

深入推进精神文明建设。着眼于培育城市精神，不断创新精神文明建设载体和内容，把全市人民的积极性、创造性凝聚到加速实现“两个率先”目标上来。着力打造以道德为支撑、产权为基础、法律为保障的“信用马鞍山”；继续开展交通秩序专项整治工作；加快实施环卫体制改革；进一步完善相对集中行政处罚权工作；全面推进摊点群自治管理，力争在全省首批跨入全国文明城市行列。

坚决维护社会稳定。深入开展以宪法为核心、市场经济法律为重点的法制宣传，不断提高市民法律意识。强化社会治安综合治理，全面实施社区警务战略，坚持“严打”方针，建立健全打防控一体化治安防控体系。认真落实安全生产责任制，加强对重大安全事故隐患的综合整治，坚决杜绝重特大事故的发生。高度重视并及时处理群众来信来访，扎实做好人民内部矛盾排查调处工作，妥善化解不稳定因素。加强国防教育，做好“双拥”工作，支持军队、民兵和人防建设。继续抓好隐蔽战线工作，切实维护国家安全。

三、奋力开创政府自身建设新局面

加速实现“两个率先”目标，对政府工作提出了新的更高要求。我们要以改革的精神进一步加强政府自身建设，努力提高施政水平。

（一）锐意改革创新，进一步提高行政工作效率。创新是事业发展的不竭动力。全体政府工作人员要自觉适应新形势下政府工作的要求，强化加快发展的意识，摒弃一切不合时宜的思想观念和行为方式，认真学习长三角等先进地区的经验，在贯彻上级文件精神与本地区本部门实际的结合上下功夫，在坚持既按章办事又不断改进工作方法上做文章，学会用改革的路子、发展的措施和市场经济的手段，化解前进中的难题和矛盾，抓住一切机遇加快发展。要鼓励创新，善待挫折，宽容失误，努力营造勇于探索、敢于创新的浓厚氛围。要加强学习型政府建设，促进政府机关人员观念不断转变和工作水平的明显提高。

（二）深化政务公开，进一步增强政府工作透明度。各级政府要继续完善政务公开制度，改进政务公开方式，让广大市民充分享有对政府事务的知情权和参与权。深入推行服务承诺制度，严格按照办事程序和时限规范操作。健全社会公共事务决策听证制度和专家咨询制度，畅通建言献策渠道，汲取民智，集思广益，提高决策的民主化、科学化水平。特别是对涉及群众利益、社会关注的重大决策和重要政策措施的出台，都要及时向社会公开。建立新闻发布制度，定期通报经济建设、城市发展、人民生活等方面的重要情况，解答群众关心的热点难点问题。继续办好市长公开电话，及时接受群众的投诉和监督。

（三）加强政风建设，进一步树立廉洁勤政的良好形象。完善目标管理体系和机关作风评议工作，严格考核奖惩，形成比实绩、比贡献、以发展论英雄的浓厚氛围。坚决克服和解决行政不作为、推诿扯皮、办事效率低下等问题，做到勇于负责，敢于决断，雷厉风行，确保政府各项工作的高效运转和各项决策部署的贯彻落实。优化政府工作规程，减少“文山会海”，加快工作节奏。讲实话、鼓实劲、做实事、求实效，不搞脱离实际、劳民伤财的“政绩工程”和“形象工程”。强化权力监督，实行“关口”前移，重点加强对权力部门的监督管理。继续完善政府采购和土地“招拍挂”等制度，推行政府投资项目集中统一管理和国库集中支付，进一步加强审计监督。加大对违法违纪案件和问题的查处力度，以反腐倡廉的实际成果树立政府的良好形象。

（四）规范行政行为，进一步推进政府工作法制化建设。即将实施的《行政许可法》，是一部规范政府行政行为的重要法律，各级政府及其部门要及时组织学习，认真贯彻执行。继续推行行政执法责任制和执法过错责任追究制，加强行政执法监督和行政复议工作。坚持向人大报告工作、向政协通报情况制度，自觉接受人大及其常委会的法律监督、工作监督和人民政协的民主监督，继续高度重视和认真办理人大代表议案、建议和政协提案。完善约见市人大代表、政协委员制度，广泛听取民主党派、工商联和社会各阶层人士的意见和建议，充分发挥工会、共青团、妇联等群众团体的桥梁纽带作用。加强基层政权和群众自治组织建设，扩大基层民主。

（五）牢记根本宗旨，进一步强化为民执政的自觉性。政府工作人员尤其是各级领导干部要不断强化“公仆”意识，树立正确的权力观、利益观和政绩观，真正做到胸怀爱民之心，多谋富民之策，善于从群众的意愿中把握工作的方向和重点。要多到群众意见多的地方去，多到工作推不开的地方去，倾听群众声音，关心群众疾苦，体察群众冷暖，为群众诚心实意办实事，尽心竭力解难事，坚持不懈做好事，努力把群众最关心的问题解决好，把群众的利益实现好、维护好、发展好。

各位代表，形势催人奋进，任务光荣艰巨。让我们紧密团结在以胡锦涛同志为总书记的党中央周围，在市委的正确领导下，深入贯彻邓小平理论和“三个代表”重要思想，全面落实党的十六大和十六届三中全会精神，以只争朝夕、奋发有为的精神状态，脚踏实地、求真务实的工作作风，同心合力，开拓进取，为加速实现“两个率先”目标而努力奋斗！
